# Local release of gemcitabine via *in situ* UV-crosslinked lipid-strengthened hydrogel for inhibiting osteosarcoma

**DOI:** 10.1080/10717544.2018.1497105

**Published:** 2018-08-27

**Authors:** Wei Wu, Yong Dai, Han Liu, Ruoyu Cheng, Qing Ni, Tingjun Ye, Wenguo Cui

**Affiliations:** a Department of General Surgery, The Affiliated Hospital of Yangzhou University, Yangzhou University, Yangzhou, P. R. China;; b Shanghai Institute of Traumatology and Orthopaedics, Shanghai Key Laboratory for Prevention and Treatment of Bone and Joint Diseases, Department of Orthopedics, Ruijin Hospital, Shanghai Jiao Tong University School of Medicine, Shanghai, P. R. China

**Keywords:** Osteosarcoma, hydrogel, *in situ*, chemotherapeutics, photocrosslinkable

## Abstract

Osteosarcoma is among the most common malignant bone tumors in human skeletal system. The conventional treatment of osteosarcoma mainly consists of combining neoadjuvant chemotherapy with surgical approach. However, it is crucial to design an artificial implant that possesses excellent biomechanical properties and is capable of sustaining local release of chemotherapeutics. In this study, we envision that the highly efficient combination of gemcitabine (GEM) hydrochloride loaded liposomes with gelatin methacryloyl (GelMA) of *in situ* photocrosslinkable hydrogel will lead to a multifunctional implant with unique antitumor, mechanical, and biodegradable properties. A sustained controlled release was observed; more specifically, the release of GEM *in vitro* lasted for 4 days long. Furthermore, its capability in killing MG63 cells was further explored by using the lixivium of GEM-Lip@Gel and GEM-GelMA hydrogel *in vitro* (composite hydrogel by GEM loaded liposomes blending with GelMA, short for GEM-Lip@Gel), which agreed with the drug release outcome. In addition, these hydrogel showed excellent ability in inhibiting osteosarcoma *in vivo* by Balb/c mice bearing MG63 cells. Therefore, GEM-loaded lipo-hydrogel certainly has presented itself as a promising strategy for the development of implant in the field of osteosarcoma treatment.

## Graphical Abstract

**Figure:**
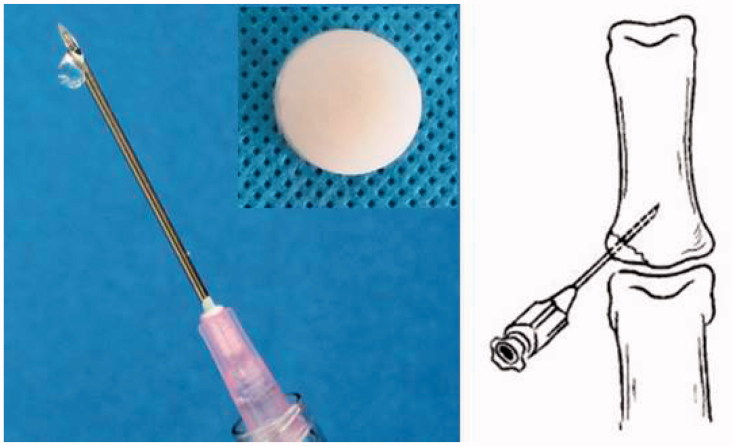

## 1. Introduction

Osteosarcoma (Ottaviani & Jaffe, 2009), also known as osteogenic sarcoma, is a type of malignant bone tumor that occurs among interosseous tissues. The cancer cells are characterized as highly malignant stromal spindle-shaped cells that are capable of immature bone or osteoid tissue formation (Rasalkar et al., 2011). Osteosarcoma is among the most common malignant bone tumors in human skeletal system. Reportedly, its incidence ranked sixth in tumors in children and adolescents, with a dismal 5-year survival rate of 65% (Longhi et al., 2006). Osteosarcoma can be broadly classified as primary (majority) and secondary (minority), with children and adolescents experiencing physical growth and development representing particularly susceptible groups (Ta et al., 2009). In addition, male morbidity rate is higher than that of the female counterpart due to the longer skeletal growth period observed in men (Fuchs & Pritchard, 2002). Typical osteosarcoma often occurs near the highly vascularized distal segments of long bones, such as distal femur and tibia; atypical osteosarcoma can still affect non-distal regions, such as skull, mandible, and vertebra (Rytting et al., 2000). The common initial symptoms of osteosarcoma include pain, swelling, and possibly local tenderness and inflammatory response; late stage symptoms include limited joint mobility and pathologic fracture. Metastasis (Liotta et al., 1991) can take place at an early stage for osteosarcoma, mainly in the lungs and bone. For 80% of the patients, micrometastasis has already taken place when they are diagnosed with osteosarcoma, which is a significant contributing factor to the low prognosis and high mortality (Strauss et al., 2010).

At present, the conventional treatment of osteosarcoma mainly consists of combining neoadjuvant chemotherapy with surgical approach (Wu et al., 2012). The chemotherapy for osteosarcoma started in the 1970s while the currently clinically available chemotherapeutics still heavily rely on doxorubicin, cisplatin, and large doses of methotrexate (Gaetano et al., 2006). In spite of playing a pivotal role in osteosarcoma treatment, the overall efficacy of chemotherapy regrettably remains around 60% due to two major limitations: high dosage induced toxic side effects and drug resistance of primary or secondary tumor cells (Morrow, 1985). With improvements in the chemotherapy efficacy, limb salvage surgery (Limb et al., 2005) has become the new treatment standard; the advent of new techniques such as inactivated local tumor replantation, bone allotransplantation, vascularized fibular grafting, and tumor-type prosthesis replacement have rendered traditional amputation obsolete (Christine et al., 2001). The key to a successful limb salvage operation lies not in tumor excision, as many would believe, but in postoperative reconstruction of bone and soft tissues. The process strives to restore the functionalities of local lesion, regain the stabilization of biomechanics, and improve the patient’s quality of life (Yazar et al., 2004). In the case of osteosarcoma, the excision area tends to be large due to the juxta-articular lesion, which in turn makes it difficult to find autologous bone in proper forms suitable for transplantation (Kabata et al., 2005). As a consequence, the reconstruction outcome is often unsatisfactory. Meanwhile, inactivation and replantation is plagued by bone fracture caused by compromised strength and not fit for cases with larger tumors. On the other hand, bone or joint allotransplantation risks high chance of rejection (Czitrom et al., 1986). Short-term speaking, prosthesis replacement has many advantages when it comes to healing and functionality; however, it suffers from long-term infection, loosening, and fracture-related issues (Roberts et al., 1991). Therefore, for the purposes of osteosarcoma treatment and bone reconstruction, it is crucial to design an artificial implant that possesses excellent biomechanical properties and is capable of sustaining local release of chemotherapeutics.

The development of material science, especially in the field of biomaterials, has attracted growing research interest due to its biocompatibility, stability, processibility, etc (Dou et al., 2013). Among them, hydrogel, a type of hydrophilic polymer that can absorb, retain, and swell but not dissolve in water, has been widely used in many biomedical applications (Dou et al., 2012). The gelation mechanism for hydrogels can be physical or chemical: physical gelation is typically a result of electrostatic interaction, hydrogen bonding, and molecular entanglement (Panda et al., 2010). Physically gelated hydrogels undergo the phase transition from gel to liquid under heat; they are mechanically weak and takes relatively longer to gelate, thus have not found many orthopedic applications (Molina et al., 2001). On the other hand, chemical gelation is typically a result of covalent crosslinking, for example, glutaraldehyde crosslinked PVA, carbon diimide crosslinked gelatin, and mercaptan double bond addition reaction (Deng et al., 2007). Common chemical crosslinking agents include genipen, adipic dihydrazide, etc; although they yield satisfactory result, the toxicity limits their spread in tissue engineering. In comparison, photocrosslinkable hydrogel demonstrates clear advantages of mild reaction, tunable production, and high biocompatibility (Yeo et al., 2007). In addition, its precursor can be directly injected subcutaneously to the lesion site, then crosslinked upon exposed to light. Moreover, constructing composite drug-loaded hydrogel by combining photocrosslinkable hydrogel with chemotherapy has huge potential in diseases’ therapy, especially in local diseases. Because local release can overcome some challenges comparing to systemic administration, such as significant side effects brought by systemic therapy, short half-life period of drugs. Thus, they are a candidate of great potential as an implant material for bone defects.

GelMA hydrogel is formed via photically or chemically induced polymerization of unsaturated methacrylate modified gelatin (Yue et al., 2015). GelMA has been utilized in many biomedical applications due to its high biocompatibility and tunable physical properties (Xin et al., 2017). Gemcitabine (GEM) is a kind of difluoronucleosides antimetabolites anticancer drugs with a broad spectrum in antitumor, the mechanism for inhibiting cancer is the destruction process of cell replication. In this study, we envision that the highly efficient combination of GEM hydrochloride loaded liposomes with GelMA will lead to a multifunctional implant with unique antitumor, mechanical, and biodegradable properties. For the *in vitro* physical characterization, tests were conducted to collect its release and mechanical profiles, as well as to verify the excellence of its abilities to control drug release, degradability, and mechanical support. In addition, the anticancer activity of the composite hydrogel was examined *in vitro*.

## 2. Materials and methods

### 2.1. Materials

The following materials are used in the study: Lecithin (CAS: 8002-43-5 from Shanghai Macklin Biochemical Co., Ltd); Cholesterol (CAS:57-88-5 from Shanghai Macklin Biochemical Co., Ltd); Gemcitabine hydrochloride (CAS:122111-03-9 from MeilunBio); Anhydrous ether (CAS:20161103 from Sinopharm Chemical Reagent Co., Ltd); 0.22 μm, 0.45 μm sterile syringe filter (from i-Quip); Gelatin (CAS:180LB8 from Rousselot Gelatin Co. Ltd); Methacrylic anhydride (CAS:760-93-0 from Aladdin); Dialysis bag (Mw: 8000–14 000, 3500 from Shanghai Yuanye Bio-Technology Co., Ltd); Centrifuge tube (Mw: 3000 from Millipore); and 2-hydroxy-4-(2-hydroxyethoxy)-2-methylpropiophenone (CAS:106797-53-9 from J&K Scientific).

### 2.2. Fabrication and characterization of GEM hydrochloride-liposome

Due to GEM hydrochloride’s hydrophilicity and low molecular weight, reverse phase evaporation method was selected for liposome fabrication (Immordino et al., 2004). About 200 mg of lecithin and 50 mg of cholesterol were first measured then dissolved in 3 mL of absolute ether and later transferred to a 250-mL rounded bottom flask. A total volume of 1 mL of GEM solution was gradually added drop by drop to the flask under a constant stirring rate of 500 rpm; the mixture then underwent 3 min of ultrasound sonication and a stable emulsion was obtained. The organic solvent was removed using a rotary evaporator at 10 °C, resulting in a gel product. About 4 mL of preheated-to-50 °C deionized water was added to the flask and the mixture was subsequently treated ultrasonically after 1 h of hydration; the ultrasound lasted for 3 min with power output set at 10%, working 2 s on and 1 s off. GEM-loaded liposome (GEM-Lip) was obtained by successive filtrations using Millipore membrane; after an adequate amount of trehalose was added as a protective agent, the liposome was freeze-dried and powder was collected in the end.

*Particle size and zeta potential*: A GEM-Lip solution of moderate concentration was prepared and its particle size distribution and zeta potential were measured using DLS (Zetasizer, Malvern, Nano-ZS90).

*Morphology*: A copper web was placed on a watch glass covered in filter papers. First 20 μL of GEM-Lip, then 20 μL of 2% phosphotungstic acid solutions were added dropwise onto the copper web surface successively with the excess being removed after 5 min each time. TEM (FEI Tecnai G-20) was used for morphology characterization. A small portion of freeze-dried GEM-Lip powder was secured by tape on a conductive copper plate and gold particles were sprayed on the plate surface. SEM (SU5000) was used to characterize the morphology of the freeze-dried GEM-Lip powder.

### 2.3. Entrapment efficiency and *in vitro* release

The entrapment efficiency and *in vitro* release of GEM-Lip were measured by the following steps: the free drug was separated from the solution using ultracentrifuge and quantified by HPLC, which served as a basis for entrapment efficiency calculations. About 1 mL of the solution was placed in the top tier and centrifuged for 5 min at 5000 rpm (Mw: 3000). Then 1 mL of deionized water was added and the mixture was centrifuged for 5 min at 5000 rpm thrice. HPLC (Shimadzu LC-2010A) was used to characterize the bottom layer of the mixture (Paolino et al., 2010). About 5 mL of GEM-Lip solution was transferred in a dialysis bag (Mw: 3500) tightened by ropes at both ends and excess rope was cut off. The dialysis bag was submerged in 35 mL of PBS solution and placed in an orbital shaker at 100 rpm for *in vitro* release at 37 °C. About 1 mL of the sample was taken from the mixture at setted time point; then 1 mL of fresh medium was replenished (Arpicco et al., 2004). The parameters for HPLC measurement were as follows: mobile phase – 0.05 M ammonium acetate, buffer – methyl alcohol (90:10), flow rate – 1 mL/min, wavelength – 268 nm, and column temperature – 30 °C.

### 2.4. Fabrication and characterization of GEM-Lip@Gel

The fabrication of GelMA (Wu et al., 2016): 20 g of gelatin was stirred and dissolved in 200 mL of PBS in an Erlenmeyer flask, which was placed in 60 °C water bath. A total volume of 16 mL methacrylic anhydride was added dropwise for duration of 1 h. Two hours later, 800 mL of preheated at 50 °C PBS was added and stirred for 15 min. The solution was then transferred into a dialysis bag (Mw: 8000–14 000). The mixture left inside the dialysis bag was collected after a week and heated to 60 °C, then filtered through a 0.22-µm microporous filtering film. The filtrate was prepared for lyophilization by prefreezing at −80 °C overnight.

The fabrication of GEM-Lip@Gel: 30, 60, and 120 mg of freeze-dried GEM-Lip powder were dissolved in 1 mL of deionized water, respectively, and 10 mg of photocrosslinking agents were added to each. Upon fully dissolving, 200 mg of GelMA (20% GelMA) were added and solutions were grouped accordingly as GEM-Lip30@Gel, GEM-Lip60@Gel, and GEM-Lip120@Gel. The prepared solutions were transferred into a mold under 6.9 mW/cm^2^d ultraviolet light (360–480 nm) for 10 s. The above steps were repeated for the 10% GelMA solutions, namely GEM-Lip30@Gel, GEM-Lip60@Gel, GEM-Lip120@Gel, and GEM-GelMA.

Morphology of GEM-Lip@Gel: SEM was used to examine the surfaces of GelMA, GEM30-Lip@Gel, GEM60-Lip@Gel, and GEM120-Lip@Gel, which were sliced and secured on a conductive copper plate, sprayed with gold on top, and characterized.

Mechanical properties of GEM-Lip@Gel: GelMA, GEM30-Lip@Gel, GEM60-Lip@Gel, and GEM120-Lip@Gel were submerged in PBS solution to swell by absorbing water at 37 °C for 24 h; they were then subject to universal testing machine compressive testing under a 1-mm/min moving condition.

Swelling and degradation properties of GEM-Lip@Gel: Freeze-dried GelMA, GEM30-Lip@Gel, GEM60-Lip@Gel, and GEM120-Lip@Gel were weighed, then submerged in PBS, taken out of the solution with surfaces wiped clean and re-weighed at setted time point. The swelling curve was plotted based on the changes in weights. GelMA, GEM30-Lip@Gel, GEM60-Lip@Gel, and GEM120-Lip@Gel were submerged in PBS at 37 °C for 24 h to let swell and weighed. Then they were submerged in hetero-collagenase II (2 U/mL) PBS solution at 37 °C and 100 rpm in a lab shaking water bath. The samples were taken out of the solution with surfaces wiped dry and re-weighed on 7, 14, 21, and 27 days. The degradation graph was plotted based on the changes in weights.

### 2.5. *In vitro* release characteristic of GEM-Lip@Gel

Both groups of GEM-Lip@Gel and GEM-GelMA were submerged in 15 mL PBS solutions in a lab shaking water bath at 37 °C and 100 rpm for *in vitro* testings. Samples were collected at set time points and measured by HPLC.

### 2.6. *In vitro* anticancer ability of GEM-Lip@Gel

*Cell viability assay*: GEM-GelMA, GEM-Lip30@Gel, GEM-Lip60@Gel, and GEM-Lip120@Gel extracts from 0–4, 4–24 , and 24–96 h were collected and used to test their effects on MG63 cell viability (Tao et al., 2017). MG63 cells (10 000 cells/well) were cultured for each group on a 96-well plate. The specific steps were outlined as follows: removing the culture medium, rinsing with PBS twice, adding 100 µL culture substrate and 10 µL CCK-8 for each sample, then placing samples in the incubator at 37 °C for 2 h, and at last measuring the absorbance values at 450 nm.

*Live/dead staining*: GEM-GelMA, GEM-Lip30@Gel, GEM-Lip60@Gel, and GEM-Lip120@Gel extracts from 0–4, 4–24, and 24–96 h were collected and used to culture MG63 cells on a 96-well plate. The culture medium was removed the next day and the fibrous lipo-gel frameworks were washed by PBS thrice. About 200 µL of the prepared dye (5 mL calcein and 20 µL ethidium homodimer in 10 mL PBS) was added to the samples and incubated at room temperature for 30 min. Then the samples were washed with the stain removal solution followed by PBS three times, and a picture was taken of each sample.

### 2.7. *In vivo* anticancer ability of GEM-Lip@Gel

The experiments were performed in accordance with the Guide for the Care and Use of Laboratory Animals from the National Institutes of Health and the protocol was approved by the Animal Care and Use Committee of Soochow University.

First, Balb/c mice were used to bear osteosarcoma as previous description, MG63 cells (1 × 10^7^) were implanted into axillary region by injection to develop humanized osteosarcoma ectopically. After 3 weeks, formed-tumor was verified by palpation, osteosarcoma-bearing mice were generally anesthetized by inhalation and the volumes (3 × 3 × 3 mm^3^) of developed tumor mass were left surgically. These surgical mice were divided into four groups randomly. The residual tumors of one group were treated with normal saline around the residual tumors as native control group and treated with GEM solution as positive control group. The ones in the other two groups were wrapped by the GEM-GelMA and GEM60-Lip@Gel hydrogel, respectively. All mice were euthanatized after 2 weeks and tumor mass was gathered, scaled, and photographed. These tumors were then fixed in 4% (v/v) paraformaldehyde solution, embedded in paraffin, sectioned into 5 μm thickness, and stained with hematoxylin and eosin (H&E Beyotime, China) for histological analysis.

### 2.8. Statistical analysis

One way ANOVA with the Tukey’s post hoc test was used to discern the statistical difference between groups. Data were presented as mean ± standard deviation. All the data were processed using IBM SPSS Statistics 22 for Windows. A probability value of *p* < .05 was considered to be statistically significant.

## 3. Results and discussion

### 3.1. Characterization of GEM hydrochloride-based liposome

**Figure 1.**
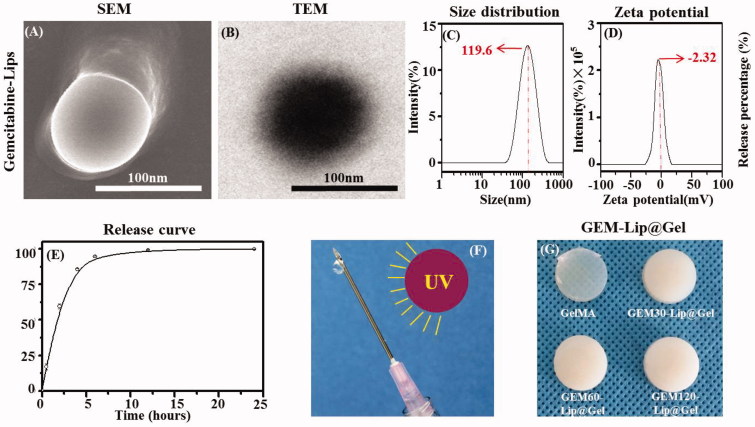
The morphology of GEM-Lip powder shown in the inset SEM images (A). Structure of GEM-loaded liposomes was shown in the TEM images in (B). The size distribution of GEM-Lip (C). Zeta potential (D). Annotation for y-coordinate was displayed in an inappropriate line (E). The mechanism of crosslinking (F). The appearance of GelMA and GEM-Lip@Gel with different content of liposome (G).

The particle size distribution, zeta potential, and morphology of GEM-Lip (Celano et al., 2004) were characterized by SEM, TEM, and DLS as shown in Figure 1. In Figure 1(A), SEM showed the liposome appeared to be round and disc-like; its diameter also matched the result from the particle size distribution graph. In Figure 1(B), TEM showed the bilamellar structure of liposomes with the lighter layer being phospholipids and the darker layer being the hydrophilic interior. Figure 1(C) showed a uniform particle size distribution with a sharp single peak. The average liposome diameter was 119.6 ± 2.3 nm. Figure 1(D) showed the zeta potential distribution with an average of −2.32 ± 0.46 mv; the slightly negative value was attributed to the unencapsulated GEM. The polydispersity index of GEM-Lip was 0.264 ± 0.031. To further characterize GEM-Lip, centrifugation ultrafiltration was utilized to isolate and remove the unencapsulated drug and the entrapment rate of 60.3 ± 2.8% was measured by HPLC. Moreover, environment stimulation and dialysis were combined to mimic and study its *in vitro* release and characteristics; the result was measured by HPLC. As demonstrated in Figure 1(E), a burst release of 85% in total was observed within the initial 4 h; the remaining 15% was essentially all released from 4 to 24 h with virtually none left after that period. Thus the *in vitro* release lasted for approximately 24 h. The noted short duration was likely a direct consequence of the low molar mass and high hydrophilicity of GEM molecules, which enabled them to take advantage of the occasional tiny space created by random motion and slip through the bilayer.

### 3.2. Morphology of GEM-Lip@Gel

**Figure 2.**
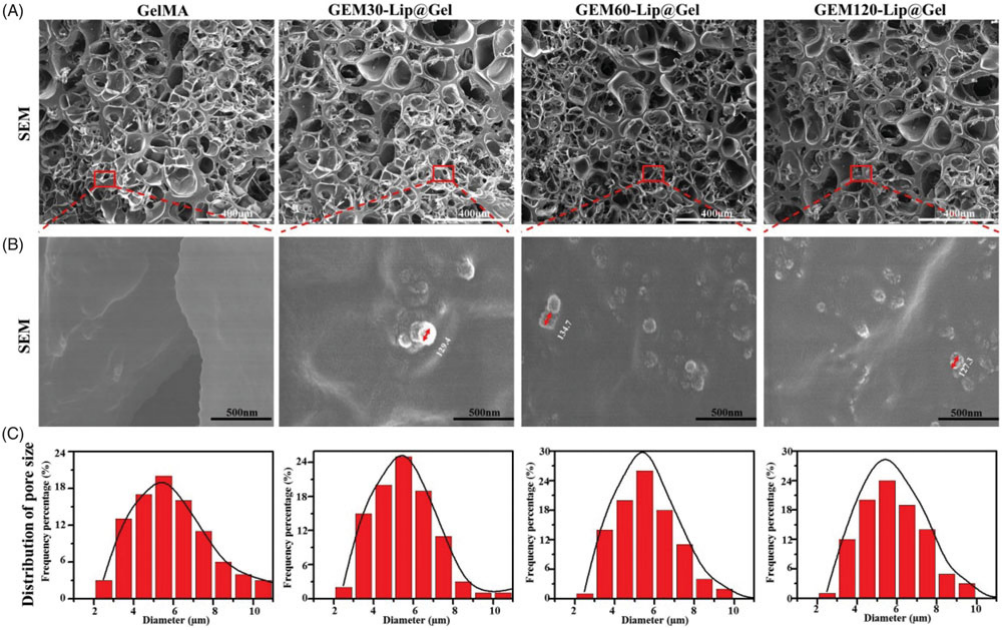
The morphology of GelMA and GEM-Lip@Gel with different quantities of liposome contents were shown in the inset SEM images in (A). The magnification of the selected area in (B). The statistics of distribution of pore size in (C).

As shown in Figure 1(F), photocrosslinking induced by ultraviolet light has been demonstrated to cause the appearance transition in GelMA from transparency to opaque (Figure 1G), and the extent of the transition varies depending on the amount of liposome added; this phenomenon is believed to be caused by the intrinsic opalescence of liposome. To verify GEM-Lip had indeed been linked and loaded to GelMA through blending, GelMA, GEM-Lip30@Gel, GEM-Lip60@Gel, and GEM-Lip120@Gel were examined under SEM. Furthermore, the influence of liposome concentration on the morphology of the composite hydrogel was investigated and is shown in Figure 2. Figure 2(A) presents the SEM micrographs of GelMA, GEM-Lip30@Gel, GEM-Lip60@Gel, and GEM-Lip120@Gel; Figure 2(B) presents the corresponding zoomed-in images of the regions enclosed by red square boxes from Figure 2(A) at a higher magnification; and Figure 2(C) presents size measurement distributions of each 100 randomly selected pores from Figure 2(A).

According to Figure 2(A), one can see that GelMA and GEM-Lip@Gels shared many similarities in the roundness of the pore, thickness of the wall, and size homogeneity. Based on Figure 2(C), the average pore sizes of GelMA, GEM-Lip30@Gel, GEM-Lip60@Gel, and GEM-Lip120@Gel were 5.08, 5.87, 5.57, and 6.04 µm respectively, confirming that variance in liposome contents had no visible impact on pore size distribution. Images in Figure 2(B) contained spherical particles of 129.4 nm for GEM-Lip30@Gel, 134.7 nm for GEM-Lip60@Gel, and 127.3 nm for GEM-Lip120@Gel, which matched the size of GEM-Lip; it was thus evident that composite hydrogels of GelMA and liposomes were formed. Because liposomes were evenly distributed in GelMA solutions, liposomal particle was noticeably sticking out on the pore surfaces of composite hydrogel.

### 3.3. Mechanical properties of GEM-Lip@Gel

To investigate the impact of GEM hydrochloride liposome content on the mechanical properties of GelMA hydrogel (Cheng et al., 2017), universal testing machine compression testing was conducted on prepared hydrogel samples.

**Figure 3.**
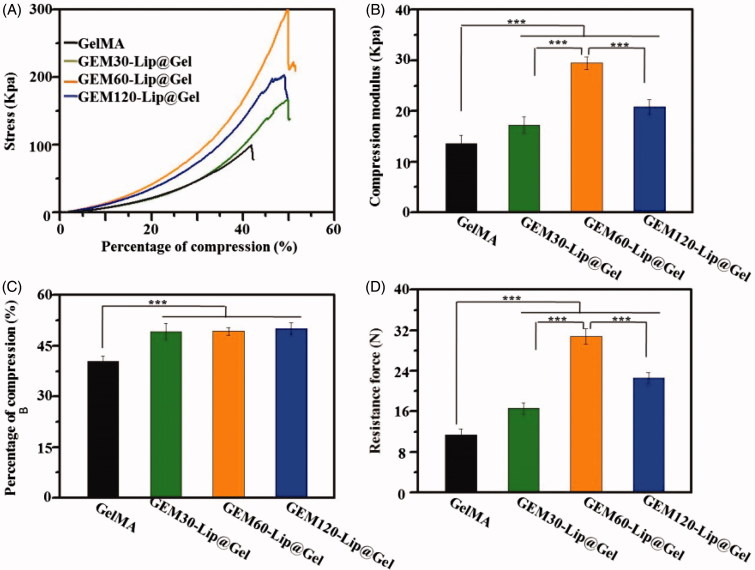
The mechanical investigation on GelMA hydrogel and GEM-Lip@Gel with different quantities of liposome contents. The compression experiments of GelMA hydrogel and GEM-Lip@Gel: (A) stress, (B) compression modulus, (C) percentage of compression, and (D) resistance force.

Figures 3(A)–(D) shows mechanical testing results from stress responsiveness, compression modulus, and compression percentage to resistance force. Compression modulus quantifies the antideformation capability of materials (Compression percentage means the percentage of deformation in hydrogel which under external force. Resistance force means the reaction force when hydrogel was compressed.). From Figure 3(B), an increase in compression modulus was observed in hydrogel samples with higher liposome content; this is supported by the fact that GEM-Lip60@Gel had a measured value of 29.49 ± 1.24 kPa, which was more than twice than that of GelMA (13.65 ± 1.53). Furthermore, Figure 3(C) clearly demonstrated that the addition of liposome noticeably enhanced compressibility from 40% in pure GelMA to 50% among composite GEM-Lip@Gel samples. Moreover, based on Figure 3(D), liposome increased the strength of hydrogel almost threefold from GelMA (11.41 ± 1.13 N) to GEM-Lip60@Gel (30.82 ± 1.49 N).

A plausible explanation for the overall mechanical property improvement is that liposomes increased the level or extent of intermolecular crosslinking through noncovalent forces, such as hydrogen bond or electrostatic interaction, by ‘wedging’ in the hydrogel grid. Hydrogen bonds can be generated between some elements in liposome, such as phosphorus, and some elements in hydrogel’s network, such as nitrogen. Those generated hydrogen bonds are able to make contribution to the mechanical property of composite hydrogel because these bonds are able to twist with hydrogel’s network to produce double crosslinking structure. For instance, when an external force was applied to the composite hydrogel, these micro-crosslinking structure can thus share and reduce external stress experienced by the framework of hydrogel itself, preserving its functionality and maximizing its usage before breaking point. The superior mechanical performance of GEM-Lip60@Gel, compared to GEM-Lip30@Gel and GEM-Lip120@Gel, can be attributed to its Goldilocks amount, a balanced compromise between not-enough and too-much liposome mixing; both are not ideal for mechanical optimization, as shown in GEM-Lip30@Gel for reasons clarified before and in GEM-Lip120@Gel for hydrophobic interaction among liposomes, which destabilized the composite hydrogel. Too-much liposome mixing will lead to fusion between these nanocarriers, which will decrease the hydrogen bone effect causing reduced mechanical property. At the same time, not-enough liposome admixture cannot generate enough hydrogen bone with hydrogel network, which also causes unsatisfactory mechanical property. The composite hydrogel can be regarded as excellent packing materials for tissue regeneration, due to its various advantages, such as noticeable capability in holding shape, stable structures, certain mechanical support, and so on. However, this kind of materials exhibit limited ability in bending, therefore, cannot be utilized as fixed devices.

### 3.4. Swelling and degradation properties of GEM-Lip@Gel

**Figure 4.**
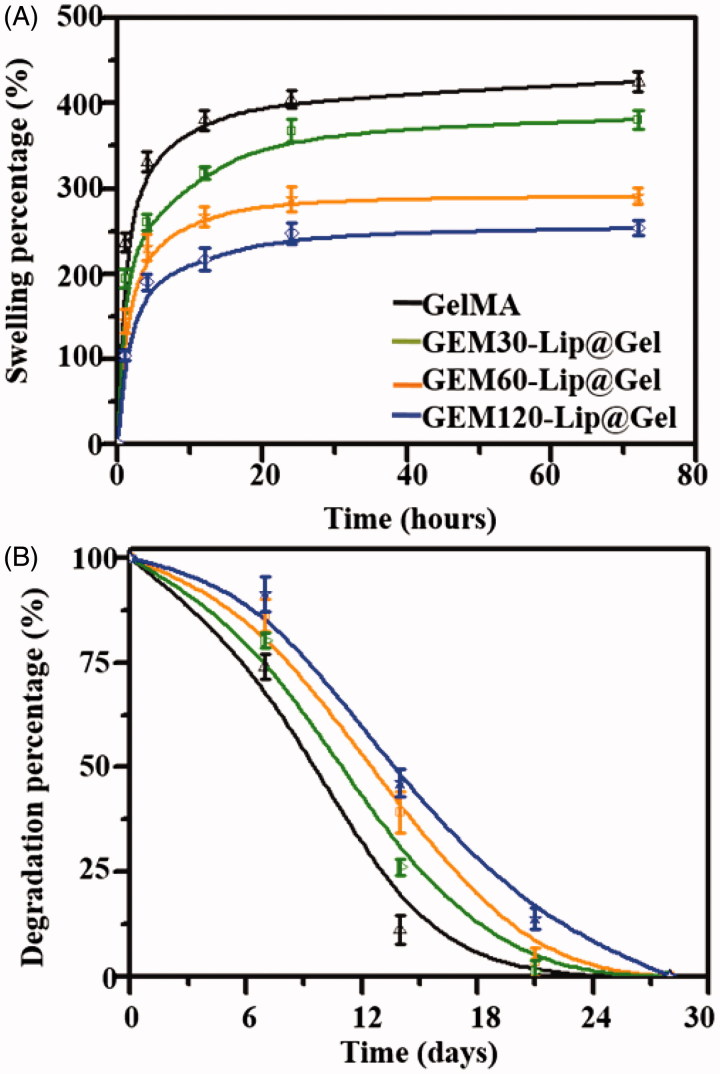
Physical characterizations of GelMA and GEM-Lip@Gel with different quantities of liposome contents. Swelling percentage (A). Degradation profiles (B).

Figure 4 highlights the capability of GelMA and GEM-Lip@Gel groups to take in medium from the outside environment (Noshadi et al., 2017). As expected, the swelling percentage showed negative correlation with the amount of liposome blended; the absorption dropped consistently from GelMA 425.31 ± 11.27% to GEM-Lip120@Gel 253.71 ± 8.87%. The low swilling ratio of GEM-Lip@Gel groups, likely caused the increased level of micro-crosslinking, is considered a desirable feature for implants as it will absorb limited liquid when contacting with fluid without significant influence in the shape of composite hydrogel.

As shown in Figure 4(B), GelMA has completely degraded by day 21, 7 days before GEM-Lip@Gel groups. On day 21, the residual mass percentages for GEM-Lip30@Gel, GEM-Lip60@Gel, and GEM-Lip120@Gel were 6.5 ± 2.1%, 9.7 ± 1.6%, and 13.9 ± 1.2% respectively. This attribute of being able to preserve the structural and functional integrity for weeks before eventual degradation is of particular importance to implants, which lays a solid foundation for future GEM-Lip@Gel implants.

### 3.5. *In vitro* release of GEM-Lip@Gel

Considering the application of functional drug-loaded hydrogel implants, a sustained release of chemotherapeutic drugs from GEM-Lip@Gel is crucial to the success of a multifunctional hydrogel, thus to osteosarcoma therapy (Wang et al., 2017). In order to further explore the ability of GEM-Lip@Gel in controlling drug release, the release profiles of GEM in GelMA and Lip@Gel groups with respective prepolymers concentrations of 10% and 20% were evaluated.

**Figure 5.**
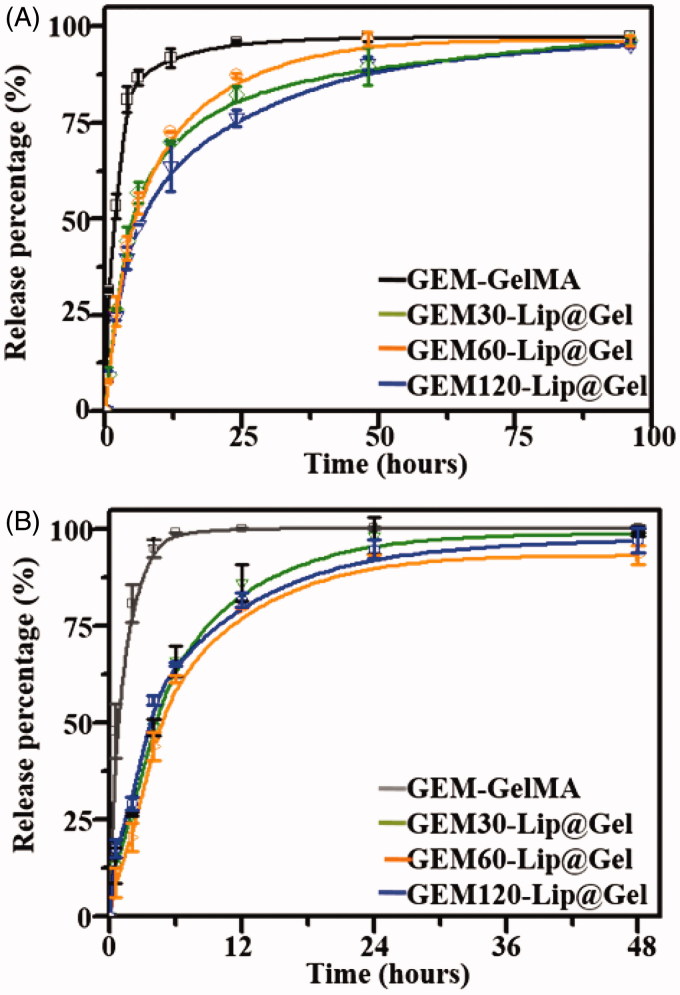
The drug release profile of GEM in GelMA and GEM-Lip@Gel with the different quantities of GEM-Lip contents at GelMA concentration of 20% (A), and at GelMA concentration of 10% (B).

Figure 5(A) shows the release profiles from all hydrogel groups fabricated from 20% GelMA and the process can be roughly divided into three phases. In Phase 1 (0–4 h), GEM-GelMA reached a cumulative release of 81.04 ± 3.37%, which was higher than its GEM-Lip@Gel counterparts of 44.36 ± 3.67%, 42.45 ± 3.03%, and 39.79 ± 3.16% for GEM-Lip30@Gel, GEM-Lip60@Gel, and GEM-Lip120@Gel, respectively. In Phase 2 (4–24 h), GEM-GelMA reached a cumulative release of 15.07%, which was lower than its GEM-Lip@Gel counterparts of 37.99%, 44.93%, and 36.45% for GEM-Lip30@Gel, GEM-Lip60@Gel, and GEM-Lip120@Gel, respectively. In Phase 3 (24–96 h), GEM@GelMA reached a cumulative release of 1.33%, which was close to nonexistent compared to its GEM-Lip@Gel counterparts of 14.0 3%, 8.86%, and 19.12% for GEM-Lip30@Gel, GEM-Lip60@Gel, and GEM-Lip120@Gel, respectively. The first stage represents the period that GEM released from GEM-GelMA was noticeably higher than that of GEM-Lip@Gel counterparts. The second stage represents the duration that GEM released from GEM-GelMA was significantly lower than that of GEM-Lip@Gel counterparts. The third stage represents the time that GEM was hardly released from GEM-GelMA, and all groups of GEM-Lip@Gel showed a more sustained release. Given the released drug of interest has a low molecular weight and is hydrophilic in nature, the release for GEM-GelMA (24 h) lasted only a quarter of its GEM-Lip@Gel counterparts (96 h) for 20% GelMA. The observed sustainability is likely due to the double barriers imposed by liposome and hydrogel scaffold; the release appeared to be less burst because of the longer diffusion time. In addition, the amount of GEM-Lip content did not significantly affect the release profile. Comparing to the half-life of 32–94 min in GEM, this composite can sustainably release medicines for about 4 days, which has significantly increased the utilization of these drugs.

Figure 5(B) shows the release profiles from all hydrogel groups fabricated from 10% GelMA and the process can be roughly divided into three phases. In Phase 1 (0–4 h), GEM-GelMA reached a cumulative release of 94.97 ± 2.20%, which was higher than its GEM-Lip@Gel counterparts of 48.77 ± 2.19%, 43.88 ± 3.61%, and 55.72 ± 1.13% for GEM-Lip30@Gel, GEM-Lip60@Gel, and GEM-Lip120@Gel, respectively. In Phase 2 (4–24 h), GEM-GelMA reached a cumulative release of 5.06%, which was close to nonexistent compared to its GEM-Lip@Gel counterparts of 49.41%, 48.92%, and 41.32% for GEM-Lip30@Gel, GEM-Lip60@Gel, and GEM-Lip120@Gel, respectively. In Phase 3 (24–48 h), no release was detected from neither GEM-GelMA nor GEM-Lip@Gel groups. The release for GEM-GelMA (6 h) lasted only a quarter of its GEM-Lip@Gel counterparts (24 h) for 10% GelMA, shorter than those of 20% GelMA. Under high concentration of GelMA, the hydrogel network is more packed and better connected, slowing down the release.

### 3.6. *In vitro* tumor inhibition of GEM-Lip@Gel

**Figure 6.**
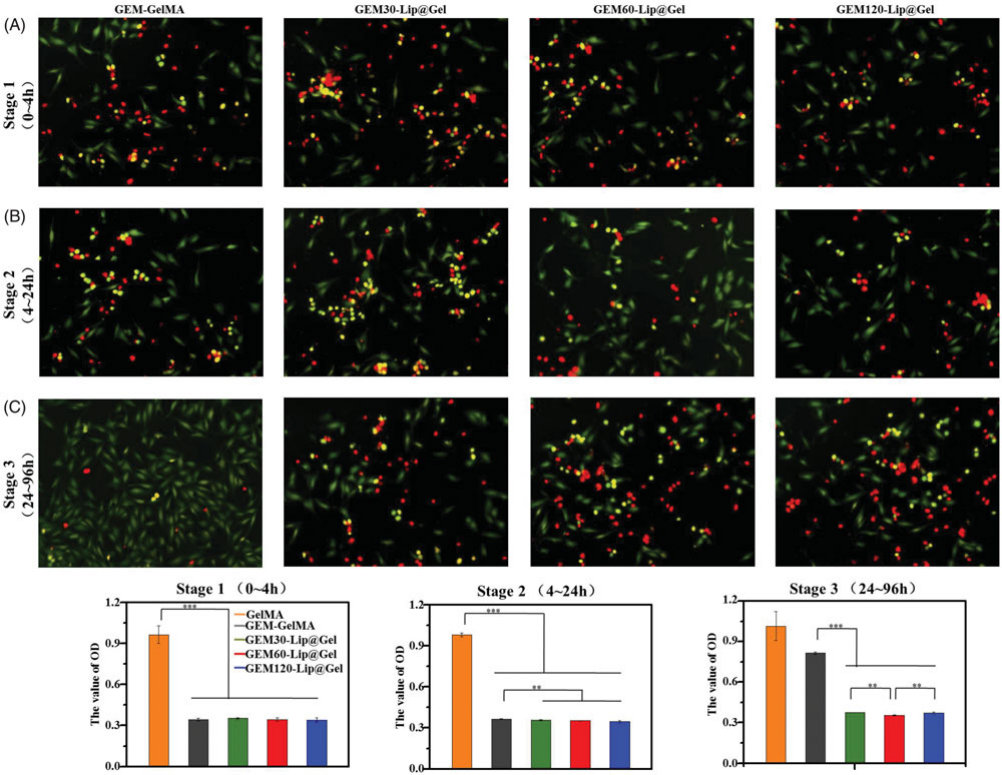
The result of CCK-8 and live/dead stain, MG63 cells were cultured with different lixivium from GEM-GelMA and GEM-Lip@Gel. The lixivium from first 4 hours, Stage 1 (A). 4–24 h, Stage 2 (B). 24–96 h, Stage 3 (C).

In order to further examine the potential of GEM-Lip@Gel functioning as an osteosarcoma inhibiting scaffold, we utilized CCK-8 and live/dead stain to investigate the capability of GEM-Lip@Gel in killing osteosarcoma *in vitro* (Popescu et al., 2017), as shown in Figure 6. The process of inhibiting osteosarcoma can be divided into three parts. During Stage 1, there was no significant difference; minimal effective concentration was achieved by all groups in spite of the fact that the release rate of GEM-Lip@Gel was slower than that of GEM-GelMA (Figure 6(A)). Because of the sustained release effect, Stage 2 is similar to Stage 1 in terms of inhibition effects for GEM-Lip@Gel. On the contrary, the inhibition effects significantly weakened in Stage 2 for GEM-GelMA (Figure 6(B)). By Stage 3, drugs in GEM-GelMA were essentially released fully. The stronger inhibiting tumor capability could be seen in the group of GEM60-Lip@Gel due to more sustained drug release capability comparing to the other two groups (Figure 6(C)). In Stage 1, there are significant differences among the GelMA groups and all groups of drug-loaded hydrogel. As for Stage 2, GEM-GelMA hydrogel shows slightly lower effect in inhibiting cancer than the other three groups of liposome modified composite hydrogel. In the Stage 3, the three groups of Lip@Gel hydrogels exhibited noticeable capability in inhibiting tumor than GelMA and GEM-Gel; moreover, among these three kinds of hydrogel, GEM-Lip60@Gel owned better effect to control ability in drug releases. It is worth mentioning that the *in vitro* results from both release profile and tumor inhibition perspectives echo each other, further supporting the optimism in GEM-Lip@Gel as postresection implants for osteosarcoma treatment.

### 3.7. *In vivo* tumor inhibition of GEM-Lip@Gel

In order to further explore the capability of GEM-Lip@Gel hydrogel in inhibiting cancer *in vivo*, osteosarcoma models were developed by injecting MG63 cells in the axillary region of Balb/c mice. After tumor masses formed, same tumor volume (3 × 3 × 3 mm^3^) was left, to be more specific, the considered residual tumor contains one-third volume of original tumor, then GEM-GelMA and GEM60-Lip@Gel hydrogel were implanted in the local residual tumor area as experimental groups. The residual tumors of one group were treated with normal saline around the residual tumors as control group. All the mice were euthanized and the tumor mass was collected 14 days after the treatment.

**Figure 7.**
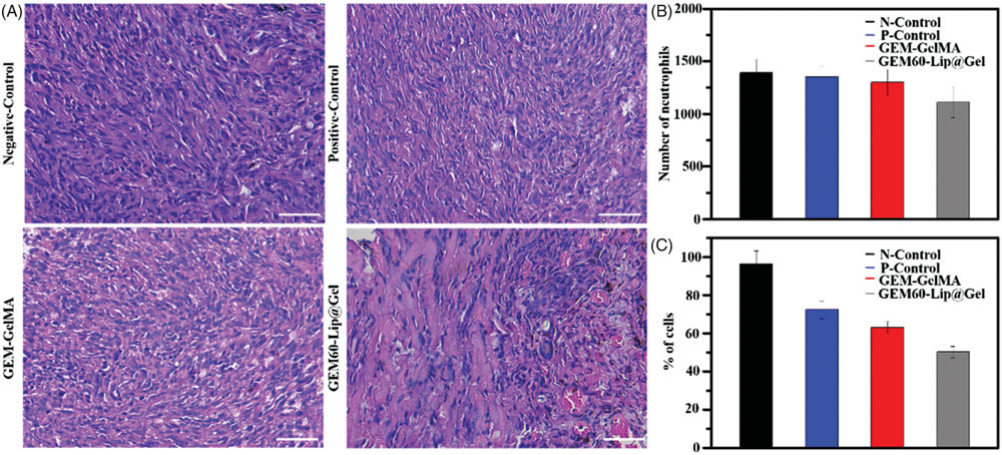
*In vivo* anticancer efficiency of GEM-GelMA and GEM-Lip@Gel hydrogel. H&E stained images (A). The number of neutrophils (B). The percentage of MG63 cells (C).

We further explored the therapeutic efficacy of GEM60-Lip@Gel hydrogel by the typical morphological assay (Figure 7(A)). H&E staining images revealed that MG63 cells were found in all groups. In the groups with the different hydrogel, nondegraded hydrogel was also observed in the tumor mass. Most MG63 cells were dead in GEM60-Lip@Gel-treated groups. The biocompatibility of composite hydrogel was further investigated by result of H&E. From Figure 7(B), it was obvious that there were no significant differences between different groups, it can prove that these functional hydrogel make limited inflammation to the tissue. In addition, the efficiency of antitumor was explored by the result of H&E. As shown in Figure 7(C), it was clear that the GEM60-Lip@Gel-treated groups have better anticancer effects than other three groups, and GEM-GelMA group slightly exhibits better result than positive group.

## 4. Conclusion

In this investigation, we developed a novel GEM-Lip@Gel scaffold capable of controlling drug release, providing mechanical support, and inhibiting osteosarcoma *in situ*. GEM-Lip was fabricated, which was a basis for further production of a liposome-modified hydrogel in controlling GEM release *in situ.* The GEM-Lip was combined with pre-crosslinking GelMA solution to construct GEM-Lip@Gel solution. After crosslinking, due to the micro-crosslinking double-network structure between liposomes and the hydrogel network, the GEM-Lip@Gel hydrogel was demonstrated to have significantly improved mechanical properties in comparison with GelMA. Moreover, a sustained controlled release was observed; more specifically, the release of GEM *in vitro* lasted for 4 days long. Furthermore, its capability in killing MG63 cells was further explored by using the lixivium of GEM-Lip@Gel and GEM-GelMA hydrogel *in vitro*, which agreed with the drug release outcome *in vitro.* Further, animal experiments exhibit the similar results *in vivo*. For the reasons mentioned above, GEM -loaded Lipo-hydrogel certainly has presented itself as a promising strategy for the development of implant in the field of osteosarcoma treatment.
